# The impact of Kangaroo Care on role adaptation and subjective well-being in primiparous mothers following vaginal delivery: A retrospective study

**DOI:** 10.1097/MD.0000000000042471

**Published:** 2025-06-27

**Authors:** Feng-Ying Li, Ming-Hua Zhong

**Affiliations:** a Maternal and Child Health Care Hospital of Xigu District, Lanzhou City, Gansu Province, China; b Department of Obstetrics and Gynecology, People’s Hospital of Xigu District, Lanzhou City, Gansu Province, China.

**Keywords:** Kangaroo Care, maternal health, primiparous mothers, role adaptation, subjective well-being

## Abstract

The postpartum period presents unique challenges for primiparous mothers, particularly in relation to role adaptation and emotional well-being. Kangaroo Care (KC), defined as skin-to-skin contact between mother and newborn, has demonstrated benefits for neonatal outcomes; however, its impact on maternal psychological health remains less extensively studied. This study aims to evaluate the effects of KC on role adaptation and subjective well-being in primiparous mothers following vaginal delivery, with the goal of identifying its potential as a supportive intervention during the maternal transition. A retrospective study was conducted between January 2020 and December 2023, enrolling 168 primiparous mothers who delivered via vaginal birth. Participants were assigned to either an observation group (n = 80), which received the KC intervention, or a control group (n = 88), which received standard postpartum care. Data were collected using a demographic questionnaire, a Role Adaptation Scale (encompassing role identification, parent–child attachment, and caregiving behavior), and a Subjective Well-Being Scale. Post-intervention scores for role adaptation and subjective well-being were compared between groups. Correlation analysis was also performed to examine the relationship between these 2 variables. Mothers in the KC group exhibited significantly higher scores across all dimensions of role adaptation: role identification (*P* = .018), parent–child attachment (*P* < .001), and caregiving behavior (*P* = .001). Additionally, KC participants reported significantly greater subjective well-being, including improvements in life satisfaction, reduced health concerns, and enhanced mood stability (all *P* < .001). Pearson correlation analysis revealed a positive association between role adaptation and subjective well-being (*R* = 0.614, *P* < .001), suggesting that enhanced adaptation is linked to better emotional outcomes. KC following vaginal delivery significantly improves both role adaptation and subjective well-being in primiparous mothers. The observed positive correlation between these outcomes highlights the potential of KC as an effective intervention for promoting maternal adjustment and emotional health in the postpartum period.

## 1. Introduction

Vaginal delivery is widely recognized as a significant milestone in a woman’s life, often accompanied by profound physiological and psychological changes. This mode of childbirth is associated with a range of maternal experiences that influence both the immediate postpartum period and the longer-term transition to motherhood. For primiparous mothers, those giving birth for the first time, this transition can present substantial challenges related to their evolving roles, emotional well-being, and overall maternal adaptation.^[1–3]^

Subjective well-being, defined as an individual’s self-evaluation of life satisfaction and emotional states, plays a critical role in shaping the postpartum experience. Studies have shown that primiparous mothers are particularly susceptible to heightened levels of anxiety, depression, and stress during the early postpartum weeks. These adverse emotional states can negatively impact maternal–infant bonding, breastfeeding success, and general health outcomes. Therefore, identifying factors that promote or hinder psychological adjustment is essential for improving maternal outcomes and quality of life during this transformative period.^[4,5]^ One intervention that has received growing attention is Kangaroo Care (KC), a practice involving direct skin-to-skin contact between mother and newborn. KC has been associated with a wide array of physiological and psychological benefits.^[6,7]^ Evidence indicates that KC can strengthen maternal–infant bonding, support breastfeeding initiation and continuation, and foster maternal confidence and competence. Moreover, KC may serve as a protective factor against postpartum mood disturbances by facilitating emotional regulation and reducing physiological stress responses. As such, it holds promise as a supportive measure for primiparous mothers undergoing the transition to motherhood.^[8,9]^

Investigating the effects of KC on role adaptation and subjective well-being among primiparous mothers is both timely and essential. With increasing emphasis on maternal mental health and the promotion of postpartum practices that enhance well-being, this study aims to explore the potential of KC in supporting maternal adjustment following vaginal delivery. By addressing this relatively under-explored area, our research seeks to contribute to the growing body of evidence advocating for effective, non-pharmacological interventions in the postpartum period.

## 2. Methods

### 2.1. Study design

A retrospective evaluation was conducted at our hospital to assess the impact of KC on role adaptation and subjective well-being in primiparous mothers following vaginal delivery. This study was carried out over a period from January 2022 to December 2023.

Inclusion criteria:

1. Mothers are primiparous (first-time mothers).
2. Parents are aged between 23 and 34 years.
3. Mothers have delivered a singleton infant via vaginal delivery, with gestational ages ranging from 37 to <42 weeks.
4. Both parents possess at least a high school diploma or vocational education, with adequate language skills for effective communication.
5. Mothers exhibit no skin lesions on the chest or abdomen and do not have severe physical illnesses or infectious diseases.

Exclusion criteria:

1. Newborns with an Apgar score of <8 at 1 minute or 5 minutes.
2. Newborns with peripheral blood glucose levels <2.6 mmol/L.
3. Newborns requiring hospitalization for pediatric treatment due to medical conditions.
4. Newborns with congenital abnormalities.
5. Incomplete data that may affect the evaluation of effectiveness and safety.

A total of 168 primiparous mothers who delivered via vaginal delivery were included as study participants. The research methodology, intent, and protocols were developed in accordance with the Strengthening the Reporting of Observational Studies in Epidemiology guidelines^[10]^ and received approval from the Ethics Committee of the People’s Hospital of Xigu District. Written informed consent for publication was obtained from all patients.

### 2.2. Assessment tools and scales

Demographic questionnaire: A customized questionnaire designed by the research team was utilized to collect general information about both the primiparous mother and father. This included demographic data such as names, ages, educational levels, occupations, income, gestational age, newborn gender, whether the pregnancy was planned, housing stress, and the status of co-parenting. Co-parenting refers to the involvement of other family members in the care of the newborn during hospitalization and the first year postpartum.

Role adaptation scale: This scale was employed to evaluate the maternal role adaptation following childbirth. It encompasses 3 dimensions: role identification, parent–child attachment, and caregiving behaviors. Each dimension consists of 8 questions scored on a 5-point Likert scale (0 to 4 points), yielding a total score range of 0 to 32 points. Scores of 26 to 32 indicate good adaptation, 20 to 25 signify moderate adaptation, and 0 to 19 reflect poor adaptation. The scale demonstrated a high reliability, with a Cronbach *α* coefficient of 0.89.

Subjective well-being scale: This scale consists of 18 items assessing various dimensions of well-being, including life satisfaction (2 items), health concerns (2 items), energy levels (4 items), mood (3 items), emotional behavior (3 items), and relaxation versus tension (4 items). Items 1 to 14 are scored on a 5- or 6-point scale, while items 15 to 18 are rated on a scale of 0 to 10. The total score ranges from 0 to 120, with higher scores indicating greater subjective well-being. This scale exhibited strong test-retest reliability (0.873) and an internal consistency coefficient exceeding 0.90.

### 2.3. Group allocation and intervention methods

From January to December 2022, the maternity ward implemented a standard nursing protocol, during which the participating mothers were classified into a control group (n = 88). In contrast, from January to December 2023, the ward adopted a KC nursing protocol, with mothers during this period assigned to an observation group (n = 80). For the control group, following the birth of the newborn, nursing staff provided routine obstetric care. The newborns were wrapped for warmth and transported to the patient room to sleep in their cribs. Mothers received standard postpartum education and guidance once daily, covering essential topics such as newborn bathing, skin-to-skin contact, umbilical cord care, breast care, breastfeeding techniques, dietary recommendations, medication management, and discharge instructions.

In the observation group, KC was integrated into the standard care approach. Nurses provided demonstrations and instructed primiparous mothers on the basic principles of newborn observation and interaction. The room temperature was adjusted to 26 to 28 °C, and necessary equipment, such as oxygen, cardiac monitors, and emergency supplies, were prepared to ensure the continuous monitoring of the newborn’s vital signs. Privacy was maintained by closing the curtains, and mothers were encouraged to wear loose-fitting clothing while comfortably reclining in a chair. The newborns were clad only in diapers and hats. Under the guidance of research personnel, mothers were assisted in positioning their naked newborns at approximately a 60-degree angle on their chests to maximize skin-to-skin contact. A soft blanket was placed over the newborn’s back, with the mother’s clothing wrapped around the infant to maintain warmth. Throughout the process, soothing background music was played to create a calm and peaceful atmosphere, fostering verbal communication and gentle touch between the mother and newborn. Research staff were present to assist and provide guidance, adjusting the newborn’s position as necessary to ensure comfort. This KC intervention was conducted twice daily during the hospitalization period (from 9:00 to 12:00 and from 14:00 to 18:00), lasting 30 to 40 minutes per session. Throughout their stay, the researchers taught mothers the techniques of KC, emphasizing important considerations. At the end of each session, mothers were encouraged to share their experiences, and affirmative language was used to validate their efforts and enhance their confidence in this nurturing practice.

### 2.4. Evaluation metrics and data collection

Data collectors guided the mothers in the ward to complete the surveys assessing their role adaptation and subjective well-being. A standardized script was used to ensure consistency in the instructions provided. A total of 168 questionnaires were distributed, and all 168 were returned, achieving a 100% response rate. The study aimed to compare the levels of role adaptation and subjective well-being in primiparous mothers at the time of their newborns’ discharge between the control and observation groups. Additionally, the correlation between role adaptation and subjective well-being was analyzed to provide insights into the potential impact of KC on maternal experiences post-delivery.

### 2.5. Statistical analysis

Statistical analyses were conducted using SPSS software (Version 27.0, IBM Corp., Armonk ). Prior to analysis, all data were categorized into quantitative or categorical variables. For quantitative variables, normality assumptions were assessed using the Shapiro–Wilk test, which is appropriate for small-to-moderate sample sizes. Variables that met the assumption of normal distribution were analyzed using independent samples *t* tests for between-group comparisons. Data are presented as mean ± SD. Categorical variables were summarized as frequencies and percentages, and between-group differences were assessed using the chi-square (*χ*²) test. When expected cell counts were insufficient, Fisher exact test was applied as an alternative. To evaluate the relationship between role adaptation and subjective well-being, Pearson correlation analysis was conducted. The strength and direction of associations were quantified using Pearson correlation coefficient (*r*). All statistical tests were 2-tailed, and a *P* value < .05 was considered statistically significant. These procedures ensured rigorous data handling and appropriate statistical inference throughout the study.

## 3. Results

### 3.1. Comparison of demographic and clinical characteristics

The baseline demographic and clinical characteristics of the observation group (n = 80) and the control group (n = 88) were generally well-matched, with no statistically significant differences observed across multiple variables. The average maternal age in both groups showed minimal variation (26.99 ± 2.44 yr in the observation group vs 27.63 ± 2.74 yr in the control group, *P* = .113). Educational attainment was also comparable, with the majority of participants in both groups holding an associate or bachelor’s degree (62.5% in the observation group and 61.4% in the control group, *P* = .666). A higher proportion of mothers in the control group had a master’s degree or above (17.0%) compared to the observation group (7.5%), though this difference was not statistically significant. Occupational distribution did not differ significantly between the 2 groups. Most participants were either employed in enterprises or were self-employed. Income levels were also similar across groups, with approximately half of each group reporting monthly incomes between 5000 and 10,000 Chinese Yuan, and a smaller percentage reporting incomes either <5000 or >10,000 Chinese Yuan.

Regarding residential area, co-parenting arrangements, and newborn gender, both groups showed similar distributions. The majority of participants resided in urban areas (55.0% in the observation group and 59.1% in the control group), and approximately half of each group reported sharing parenting responsibilities with another family member. Newborn gender was nearly evenly distributed in the observation group, while the control group had a slightly higher proportion of male infants, though these differences were not statistically significant (*P* > .05). In terms of clinical characteristics, gestational age at delivery was consistent between groups, with mean gestational weeks of 38.58 ± 1.02 in the observation group and 38.76 ± 0.95 in the control group (*P* = .238). The presence of health insurance was also comparable, with over 80% of participants in each group reporting insurance coverage (*P* = .309). Planned pregnancies were similarly prevalent across both groups, with >79% of participants in each group having planned pregnancies (*P* = .974) (Table 1). Overall, the lack of significant differences in demographic and clinical characteristics between the observation and control groups suggests a well-balanced sample, minimizing potential confounding variables and enhancing the reliability of subsequent analyses.

**Table 1:** Comparison of demographic and clinical characteristics.

Item	Observation group (n = 80)	Control group (n = 88)	*t*/*χ*² value	*P* value
Mother’s age (yr, $\bar{x}\pm s$)	26.99 ± 2.44	27.63 ± 2.74	1.593	.113
Education level (n [%])			0.812	.666
High school or vocational	24 (30.0%)	19 (21.6%)		
Associate or Bachelor	50 (62.5%)	54 (61.4%)		
Master or above	6 (7.5%)	15 (17.0%)		
Occupation (n [%])			1.667	.197
Enterprise employee	38 (47.5%)	45 (51.1%)		
Civil servant	3 (3.8%)	4 (4.5%)		
Self-employed	20 (25.0%)	22 (25.0%)		
Other	19 (23.8%)	17 (19.3%)		
Income (n [%])			1.554	.122
3000-5000 CNY/month	9 (11.3%)	7 (8.0%)		
5000-10,000 CNY/month	43 (53.8%)	45 (51.1%)		
>10,000 CNY/month	28 (35.0%)	36 (40.9%)		
Residence area (n [%])			0.286	.593
Urban	44 (55.0%)	52 (59.1%)		
Rural	36 (45.0%)	36 (40.9%)		
Co-parenting (n [%])			0.149	.700
Yes	44 (55.0%)	51 (58.0%)		
No	36 (45.0%)	37 (42.0%)		
Newborn gender (n [%])			0.76	.383
Male	41 (51.3%)	51 (58.0%)		
Female	39 (48.8%)	37 (42.0%)		
Gestational weeks (wk, $\bar{x}\pm s$)	38.58 ± 1.02	38.76 ± 0.95	1.184	.238
Health insurance (n [%])			1.034	.309
Yes	70 (87.5%)	72 (81.8%)		
No	10 (12.5%)	16 (18.2%)		
Planned pregnancy (n [%])			0.001	.974
Yes	65 (81.3%)	70 (79.5%)		
No	15 (18.8%)	18 (20.5%)		

CNY = Chinese yuan.

### 3.2. Role adaptation scale scores

The role adaptation scale scores, evaluating aspects such as role identification, parent–child attachment, and caregiving behavior implementation, were significantly higher in the observation group that received the KC intervention compared to the control group, indicating the positive impact of KC on role adaptation. For role identification, the observation group achieved a significantly higher mean score (29.09 ± 2.76) than the control group (27.95 ± 3.38), with a statistically significant difference (*P* = .018). This suggests that mothers in the observation group developed a stronger and more confident sense of their maternal role through the support of KC. Parent–child attachment scores were also notably improved in the observation group, which scored 28.94 ± 3.14 compared to 26.94 ± 3.37 in the control group (*P* < .001). This finding implies that KC promoted a closer emotional bond between mother and infant, a critical element in the early stages of motherhood (Table 2).

**Table 2:** Role adaptation scale scores of observation and control groups (mean ± SD).

Item	Observation group (n = 80)	Control group (n = 80)	Mean difference (95% CI)	*t* value	*P* value	Cohen *d*
Role identification	29.09 ± 2.76	27.95 ± 3.38	1.14 (0.18–2.10)	2.380	.018	0.37
Parent-child attachment	28.94 ± 3.14	26.94 ± 3.37	2.00 (0.98–3.02)	3.968	<.001	0.61
Caregiving behavior implementation	27.09 ± 4.29	24.21 ± 3.78	2.88 (1.62–4.14)	4.625	.001	0.71
Total score	85.28 ± 9.08	79.18 ± 8.99	6.10 (3.28–8.92)	4.372	<.001	0.68

In terms of caregiving behavior implementation, mothers in the observation group again showed higher scores (27.09 ± 4.29) compared to the control group (24.21 ± 3.78, *P* = .001). This reflects enhanced maternal competence and confidence in caregiving tasks, likely supported by the skin-to-skin contact and active engagement components of KC. The total score on the role adaptation scale, encompassing all measured dimensions, was significantly higher for the observation group (85.28 ± 9.08) compared to the control group (79.18 ± 8.99, *P* < .001). This overall improvement highlights the effectiveness of KC in facilitating maternal adaptation to the caregiving role (Table 2). In summary, these results suggest that the KC intervention significantly enhanced role adaptation across multiple domains, fostering a smoother transition to motherhood and strengthening the mother–infant bond in the observation group. This intervention may thus be a valuable approach in supporting new mothers during the postpartum period.

### 3.3. Antimicrobial resistance profiles of major pathogens involved in infection

The subjective well-being scores, encompassing dimensions such as life satisfaction, health concerns, energy, mood, emotional behavior, and relaxation, demonstrated notable improvements in both the observation and control groups post-intervention. However, the gains were significantly greater in the observation group, highlighting the effectiveness of KC in enhancing maternal well-being. In the observation group, there was a marked increase in subjective well-being across all dimensions following the KC intervention. Notable improvements were observed in life satisfaction (pre-intervention: 6.93 ± 1.72, post-intervention: 7.89 ± 1.43, *P* < .001), health concerns (from 5.93 ± 2.15 to 8.01 ± 1.82, *P* < .001), and energy levels (from 16.93 ± 2.69 to 20.39 ± 3.07, *P* < .001). These results suggest that KC had a substantial impact on reducing maternal stress and enhancing physical vitality. Additionally, depression or positive mood scores improved significantly, from 15.18 ± 2.11 to 19.81 ± 1.92 (*P* < .001), indicating enhanced mood stability and reduced negative affect in mothers who practiced KC. The total subjective well-being score in the observation group rose from 74.16 ± 6.84 pre-intervention to 89.68 ± 6.38 post-intervention (*P* < .001), underscoring the comprehensive positive effects of the KC intervention (Table 3). This increase reflects an overall improvement in the subjective well-being of mothers, which is vital for the postpartum adjustment phase.

**Table 3:** Subjective well-being scale scores of observation and control groups.

Group	Time point	Life satisfaction	Health concerns	Energy	Depression or positive mood	Emotional behavior	Relaxation vs tension	Total score
Observation group (n = 80)	Pre-intervention	6.93 ± 1.72	5.93 ± 2.15	16.93 ± 2.69	15.18 ± 2.11	12.16 ± 2.10	16.89 ± 3.58	74.16 ± 6.84
	Post-intervention	7.89 ± 1.43	8.01 ± 1.82*	20.39 ± 3.07*	19.81 ± 1.92*	14.74 ± 1.84*	19.03 ± 2.96	89.68 ± 6.38*
	*t* value	3.946	6.787	7.736	14.89	8.486	4.237	15.22
	*P* value	<.001	<.001	<.001	<.001	<.001	<.001	<.001
Control group (n = 88)	Pre-intervention	7.01 ± 1.48	6.03 ± 1.75	16.12 ± 2.04	16.61 ± 3.09	12.65 ± 1.62	16.63 ± 2.37	75.85 ± 5.10
	Post-intervention	7.63 ± 1.68	6.69 ± 1.42	18.36 ± 2.98	18.08 ± 2.62	13.97 ± 1.98	18.41 ± 2.82	82.43 ± 7.26
	*T* value	2.527	2.747	5.819	3.404	4.840	4.533	6.957
	*P* value	.012	.007	<.001	<.001	<.001	<.001	<.001

Compared with the control group post-intervention:

* *P* < .05.

In the control group, modest improvements were observed across several well-being dimensions post-intervention. For example, life satisfaction showed a minor increase from 7.01 ± 1.48 to 7.63 ± 1.68 (*P* = .012), and health concerns scores increased from 6.03 ± 1.75 to 6.69 ± 1.42 (*P* = .007). However, these changes were comparatively smaller than those in the observation group (Table 3). While the control group also experienced improvements in energy, mood, and relaxation versus tension, these were less pronounced, indicating that standard care alone had limited effects on enhancing maternal well-being. Overall, the results indicate that the KC intervention led to significantly greater improvements in subjective well-being for mothers in the observation group compared to those receiving standard care.

### 3.4. Correlation analysis between subjective well-being and role adaptation in primiparous mothers

Pearson correlation analysis demonstrated a significant positive relationship between subjective well-being and role adaptation levels in primiparous mothers (*R* = 0.614, *P* < .001). This suggests that as mothers experience higher subjective well-being, their adaptation to the maternal role is also enhanced, indicating an interdependent relationship between emotional well-being and role adjustment during the postpartum period. Further analysis of the individual dimensions within role adaptation revealed that all 3 key components – role identification, parent–child attachment, and caregiving behavior implementation – were positively correlated with subjective well-being. Specifically, role identification showed a strong positive correlation with subjective well-being (*R* = 0.563, *P* < .001), suggesting that mothers who felt a stronger sense of identity in their new maternal role also reported higher levels of well-being. Similarly, parent–child attachment was positively correlated with well-being (*R* = 0.533, *P* < .001), indicating that a stronger bond with the newborn enhances maternal emotional satisfaction. Lastly, caregiving behavior implementation also demonstrated a significant positive correlation (*R* = 0.477, *P* < .001), emphasizing the role of hands-on caregiving in contributing to maternal well-being.

### 3.5. Post hoc power analysis

A post hoc power analysis was performed to assess the adequacy of the sample size in detecting the observed effects. Statistical power was calculated for each of the key outcome measures: role identification, parent–child attachment, caregiving behavior, total role adaptation score, and total subjective well-being score. To derive a global sensitivity index, we calculated a weighted overall power using the non-centrality parameter associated with each effect as the weight. The computed overall power was 0.96, indicating that the sample size was more than sufficient to detect the observed medium-to-large effect sizes with a high degree of statistical reliability. This further supports the robustness of the findings despite the retrospective design.

## 4. Discussion

The postpartum period is a critical time for primiparous mothers as they undergo substantial physical, emotional, and psychological adjustments while assuming their new maternal role. This transition often involves challenges such as role adaptation, in which mothers develop a sense of identity and confidence in their caregiving abilities, as well as subjective well-being, which is vital for emotional stability and overall quality of life.^[11,12]^ KC – a practice involving skin-to-skin contact between mother and infant – has been widely studied for its benefits in neonatal care, particularly in enhancing bonding and stabilizing the infant’s physiological responses. However, increasing attention has been given to its potential effects on maternal outcomes, such as role adaptation and subjective well-being. KC offers a unique, hands-on caregiving approach, fostering intimate physical and emotional connection between mother and infant.^[13,14]^ The findings of this study underscore the significant positive impact of KC on both role adaptation and subjective well-being in primiparous mothers following vaginal delivery. By examining scores across multiple domains of these constructs, our results highlight KC as a promising intervention for promoting maternal psychological and emotional health during the postpartum period.

KC intervention was associated with significant improvements across all dimensions of role adaptation, including role identification, parent–child attachment, and caregiving behavior implementation. A plausible mechanism underlying these findings lies in the core components of KC – skin-to-skin contact and physical proximity – which not only enhance maternal role awareness but also strengthen confidence in that role. Skin-to-skin contact has been shown to stimulate oxytocin release, a hormone that facilitates maternal behaviors and bonding with the infant.^[15,16]^ This physiological response may account for the higher role identification scores observed in the KC group, as mothers may have felt more emotionally connected and assured in their ability to care for their newborns.

Moreover, the emphasis on sustained physical closeness and interactive engagement in KC likely contributed to the significantly higher parent–child attachment scores in the intervention group. Early attachment is critical in the postpartum period and is associated with favorable long-term developmental outcomes. Skin-to-skin contact has been linked to enhanced maternal sensitivity and responsiveness to infant cues, which may help explain the improved attachment outcomes. These findings are consistent with prior research suggesting that physical closeness promotes maternal–infant bonding, reinforcing the potential of KC as a mechanism to foster early attachment.^[17,18]^ The observed enhancement in caregiving behavior implementation further supports the role of KC in promoting maternal self-efficacy and caregiving competence. Through direct and active involvement in newborn care, mothers may develop a greater sense of capability and confidence, thereby reducing anxiety associated with caregiving responsibilities and fostering a sense of accomplishment. KC encourages maternal engagement in nurturing activities, which can improve preparedness and confidence in managing the multifaceted demands of infant care.^[19,20]^ These findings support the hypothesis that structured, interactive interventions such as KC can facilitate role adaptation by enhancing both emotional connection and practical caregiving skills.

Beyond its impact on role adaptation, KC also demonstrated a substantial effect on subjective well-being, encompassing dimensions such as life satisfaction, health concerns, energy, mood, emotional behavior, and relaxation. The significant improvements observed among mothers in the KC group suggest that this intervention plays an important role in addressing the emotional and psychological needs of postpartum women. One plausible explanation is that KC exerts a calming effect through sustained skin-to-skin contact, which may reduce maternal stress and anxiety. This aligns with existing evidence indicating that physical closeness with the infant can lower cortisol levels, thereby reducing stress and contributing to a more positive emotional state.^[21,22]^ Notably, energy levels and mood stability were enhanced in mothers practicing KC, as reflected by higher scores in domains such as energy, positive mood, and reduced depressive symptoms. The intimate physical contact and interaction inherent to KC may foster a sense of emotional fulfillment and satisfaction, helping to alleviate fatigue and mood disturbances commonly experienced during the postpartum period. Moreover, the supportive nature of KC – including its emphasis on physical touch and verbal interaction – likely contributes to greater emotional balance and reduced psychological distress. These improvements are clinically relevant, as maternal well-being directly influences the quality of caregiving and the overall mother–infant relationship.

The strong positive correlation observed between subjective well-being and role adaptation in this study suggests an interdependent relationship between these 2 constructs. Specifically, higher levels of subjective well-being were associated with greater maternal role identification, stronger parent–child attachment, and more effective caregiving behavior implementation. This finding implies that emotionally stable and satisfied mothers are more likely to embrace their maternal role and confidently engage in caregiving activities. Conversely, successful adaptation to the maternal role may enhance well-being by reducing stress and fostering a sense of purpose and accomplishment.^[23,24]^ Further analysis of individual dimensions supports this association. Role identification demonstrated a particularly strong correlation with subjective well-being, suggesting that a clear and confident maternal identity contributes significantly to emotional health. Similarly, parent–child attachment was positively correlated with well-being, underscoring the psychological benefits of early bonding. Finally, caregiving behavior implementation was associated with improved emotional states, likely due to reduced caregiving-related anxiety and increased maternal self-efficacy.^[25,26]^

Recent studies have provided valuable insights into the physiological and emotional benefits of skin-to-skin care for mothers and newborns. Maleki et al’s^[27]^ study demonstrates that nursing strategies such as skin-to-skin care and family-centered interventions significantly reduce maternal stress in neonatal intensive care settings. Similarly, our study highlights that KC fosters role adaptation and subjective well-being. While both approaches emphasize skin-to-skin contact, our findings extend the evidence to primiparous mothers after vaginal delivery, strengthening the understanding of KC’s broader psychological benefits. Martínez-Rodríguez et al^[28]^ provide strong evidence that skin-to-skin contact benefits maternal outcomes, including reduced postpartum hemorrhage risk and improved uterine health during the third stage of labor. In contrast, our research focuses on psychological outcomes, revealing enhanced role adaptation and subjective well-being. Together, these findings highlight KC’s multifaceted impact, supporting its integration into postpartum care protocols. Clarke-Sather et al’s^[29]^ study reports physiological advantages of skin-to-skin contact during labor’s third stage, such as reduced need for uterotonics and quicker placental expulsion. Our study, however, shifts the lens toward psychological outcomes, demonstrating improvements in role adaptation and well-being. By complementing these physiological insights, our work underscores the holistic value of KC, emphasizing its role in maternal emotional health postpartum.

This study provides valuable insights into the impact of KC on role adaptation and subjective well-being in primiparous mothers; however, several limitations must be acknowledged. First, the retrospective design limits our ability to establish causality, and while we attempted to mitigate biases through rigorous statistical analysis, unmeasured confounders, such as maternal mental health history, social support, and breastfeeding practices, may still influence the findings. Future prospective studies should address these factors to better elucidate the underlying mechanisms through which KC affects maternal outcomes. Additionally, the study’s single-institution setting limits the generalizability of the results to diverse healthcare environments. Multi-center and cross-cultural studies are needed to confirm the broader applicability of our findings and to explore how cultural and healthcare system differences may influence KC outcomes. The relatively short observation period in this study restricts our understanding of the long-term effects of KC. Longitudinal studies tracking mothers over extended postpartum periods would provide a more comprehensive view of whether the positive effects observed in the immediate postpartum period persist over time. Furthermore, the reliance on self-reported measures introduces the potential for subjective bias. Future research could benefit from incorporating both objective measures and self-reports to provide a more robust understanding of KC’s impact on maternal well-being.

## 5. Conclusions

KC following vaginal delivery significantly enhances role adaptation and subjective well-being in primiparous mothers. The positive correlation between subjective well-being and role adaptation suggests that better role adjustment is associated with higher well-being. These findings support KC as a valuable intervention to improve maternal adjustment and emotional health in the postpartum period.

## Acknowledgments

We thank every patient and staff who participated in this study.

## Author contributions

**Conceptualization:** Feng-Ying Li, Ming-Hua Zhong.

**Data curation:** Feng-Ying Li.

**Formal analysis:** Feng-Ying Li.

**Investigation:** Feng-Ying Li.

**Methodology:** Feng-Ying Li, Ming-Hua Zhong.

**Resources:** Feng-Ying Li.

**Software:** Feng-Ying Li.

**Writing – original draft:** Feng-Ying Li.

**Writing – review & editing:** Ming-Hua Zhong.
